# Words Don't Come Easy: How Male Prisoners' Difficulties Identifying and Discussing Feelings Relate to Suicide and Violence

**DOI:** 10.3389/fpsyt.2020.581390

**Published:** 2020-12-10

**Authors:** Laura Hemming, Peer Bhatti, Jennifer Shaw, Gillian Haddock, Daniel Pratt

**Affiliations:** ^1^Division of Psychology and Mental Health, School of Health Sciences, University of Manchester, Manchester, United Kingdom; ^2^Manchester Academic Health Sciences Centre, Manchester, United Kingdom; ^3^Greater Manchester Mental Health NHS Foundation Trust, Manchester, United Kingdom

**Keywords:** alexithymia, suicide, aggression, emotion dysregulation, forensic, violence

## Abstract

Suicide and violence are prevalent within male prisons in the UK. It has been suggested that alexithymia may be associated with both suicide and violence. Alexithymia can be defined as an inability to identify or discuss emotions. The present study aimed to qualitatively explore male prisoners' experiences of alexithymia and how these experiences may relate to suicide and violence. Fifteen male prisoners were recruited from two prisons in the North West of England. All participants had experienced suicidal and/or violent thoughts and/or behaviors in the past 3 months, and all screened positive on an established measure of alexithymia. Participants took part in a qualitative interview during which they were also given the opportunity to provide drawings of their emotions. Data were transcribed and analyzed using thematic analysis, with a collaborative approach taken between researchers and an individual with lived experience of residing in prison. The results indicated that male prisoners tended not to talk about their emotions with others, due to external pressures of residing in prison in addition to internal difficulties with recognizing and articulating emotions. Not discussing emotions with others was associated with a build-up of emotions which could result in either an emotional overload or an absence of emotions. Both experiences were perceived to be associated with hurting self or others, however, participants also identified a “safety valve” where it was acknowledged that using more adaptive approaches to releasing emotions could prevent harm to self and others. These findings suggest three main clinical implications; (1) a cultural shift in male prisons is needed which encourages open communication of emotions (2) individualized support is needed for those identified as experiencing a difficulty in recognizing and articulating emotions and (3) prison staff should encourage alternative ways of releasing emotions such as by using harm minimization or distraction techniques.

## Introduction

Suicide and violence present a major issue in UK prisons. In prisons across England and Wales in the year 2019, there was a total of 80 self-inflicted deaths (1 per 1,000 prisoners), and 63,328 self-harm incidents (156 per 1,000 prisoners) (1). Furthermore, in the same year, there was a total of 32,669 assault incidents, of which 3,813 were serious assaults (46 per 1,000 prisoners) (1).

It is frequently suggested that both suicide and violence are related to difficulties with emotion regulation. For instance, it's proposed that suicide stems from unmanageable feelings with which one is unable to cope (2, 3), and that violence stems from an inability to regulate emotions such as anger (4, 5). One specific form of emotion dysregulation which has been found to relate to both suicide and violence is alexithymia. Alexithymia can be defined as an inability to identify or communicate emotions (6). Alexithymia has been found to be related to suicide and self-harm in community populations (7, 8). Furthermore, a link has been established between alexithymia and violence both in community populations (9–13) and in offenders (14, 15).

To date, there has been little qualitative research which has explored either the experiences of alexithymia in prisoners or the relationship between alexithymia, suicide and violence. Of note, Laws and Crewe (16) explored how and why prisoners regulate their emotions. They reported that the majority of prisoners felt that open displays of emotion were hazardous. This was due to threat of punishment (due to showing aggressive emotions) or exploitation from other prisoners (due to showing “vulnerable” emotions such as sadness and pain). As a result, prisoners reported only sharing their intimate feelings with loved ones via phone calls, visitations and letter writing. As a result of not having an avenue to release these emotions, prisoners reported harming themselves as a way to release negative emotions. In another qualitative study, Hemming et al. (17) explored prison staff's perceptions of the relationship between alexithymia, suicide and violence in male prisoners. Staff in this study reported that prisoners “don't do feelings,” stating that they perceived prisoners to experience difficulties with identifying, understanding and communicating feelings. These difficulties were thought to lead to intense, sudden outbursts of emotion, which were managed either by hurting self or others or through using drugs and alcohol. This process was placed within the context of limited opportunity to learn about emotions when growing up, and also within the current context of prison whereby the environment necessitated not discussing emotions.

Due to the dearth of previous qualitative research on the experiences of alexithymia and how these relate to harmful behaviors in a prison population, this study sought to explore these issues in more detail. Specifically, the two main research questions were;

1. How do male prisoners experience alexithymia?
2. How does alexithymia relate to suicide and violence in male prisoners?

## Materials and Methods

### Ethics Statement

This study was reviewed and approved by NHS England Research Ethics Committee (17/NE/0132) and HMPPS (2017-268). Participants provided their written informed consent to participate in this study. Written informed consent was obtained from the individuals for the publication of any potentially identifiable data, including images or quotes, included in this article.

### Sampling

Participants were eligible to take part in this qualitative study if they were aged 18 or over and were residing in a prison in North West of England for at least 1 week. Participants must have experienced custodial suicidal and/or violent thoughts and/or behaviors in the past 3 months, and score 52 or above on the Toronto Alexithymia Scale (18). Participants were excluded from the study if they; did not possess sufficient English language skills to take part in the interview; did not possess sufficient mental capacity to provide informed consent; were assessed by the prison's security department and deemed too high risk due to security intelligence to move around the estate or be seen by a researcher; were due for a release or transfer in the next 1 week. Purposive maximum variation sampling was used to recruit prisoners who varied across key dimensions including alexithymia score, whether the individual had experienced suicide and/or violence or both whilst in custody, age and ethnicity.

Eligibility was assessed by a researcher (LH) administering the Toronto Alexithymia Scale (18) and by asking four further screening questions to determine eligibility: (1) Over the past 3 months have you thought about killing yourself? (2) Over the past 3 months have you tried to kill yourself? (3) Over the past 3 months have you thought about hurting somebody else? (4) Over the past 3 months have you tried to hurt somebody else? Participants either self-referred to the study and were asked these eligibility questions, or they were assessed as eligible by a researcher (LH) based on their participation in a separate cross-sectional study Hemming et al., in submission which examined the relationship between alexithymia and harm to self or others.

Prisoners were invited to a one-to-one face-to-face meeting with a researcher (LH) where the information sheet was presented and discussed. Prisoners were then given a minimum of 24 h to consider their participation in the study. Participants were recruited from two prisons in the North West of England; one of which was a category A local prison, and one a category C. Fifteen participants were recruited at which point data sufficiency was felt to be reached (19); the research team perceived that no new themes were being generated from the data. A further five participants declined to take part in a research interview, with reasons for non-participation including fears that the audio recording may be shared with others, that participation would negatively affect their parole and having “too much going on” at that time to take part in an interview.

### Data Collection

Participants were asked to complete a demographic questionnaire. A flexible, open-ended topic guide, which was pilot tested with members of a patient and public involvement (PPI) group, was used to explore prisoners' experiences of: strong emotional experiences and expression, emotional upbringing and learnings from this, recent experiences of suicidal/violent thoughts and/or behaviors and the relationship between emotional experiences and suicide/violence. During data collection, the topic guide evolved to require participants be offered the opportunity to draw their emotions. Participatory drawing as a visual research method has previously been identified as beneficial due to its lack of dependence on linguistic proficiency (20). This was particularly relevant in the present study due to participants being recruited into the study for their lack of proficiency in articulating their emotions, as indicated by a high score on the TAS-20. Of the eleven participants that were given the opportunity to create drawings as part of the interview, nine did so. These participants created between one and four drawings each, creating a total of 19 drawings. All interviews were conducted by LH on a one-to-one basis, without any prison staff immediately present, in a location within the host prisons between May 2018 and November 2019. Interviews were audio-recorded and transcribed verbatim and identifying information was removed from transcripts and drawings. Interviews lasted on average 61 min (range 37–88 min).

### Analysis

The data were analyzed using inductive thematic analysis to identify common themes and discrepancies across the accounts of participants (21). Additionally, a polytextual thematic analysis was conducted on the drawings that participants had created (22). First, researchers familiarized themselves with the data. This was achieved by transcribing the interviews, and by looking over both transcripts and drawings whilst making reflective notes. Transcripts were then coded inductively and drawings were coded deductively using the coding framework developed from transcript data. Codes were then sorted into themes collaboratively with a patient and public contributor (PB).

### Patient and Public Involvement

A dedicated group of ex-offenders was recruited to assist with several tasks throughout the lifecycle of this study. This group advised on recruitment strategies, time and place to conduct interviews, the interview procedure, the topic guide, tone of the interview, how to manage distress and disclosure of intent to harm during an interview and with dissemination of the findings including assisting with the writing of this paper and producing a participant summary.

**Figure 1:**
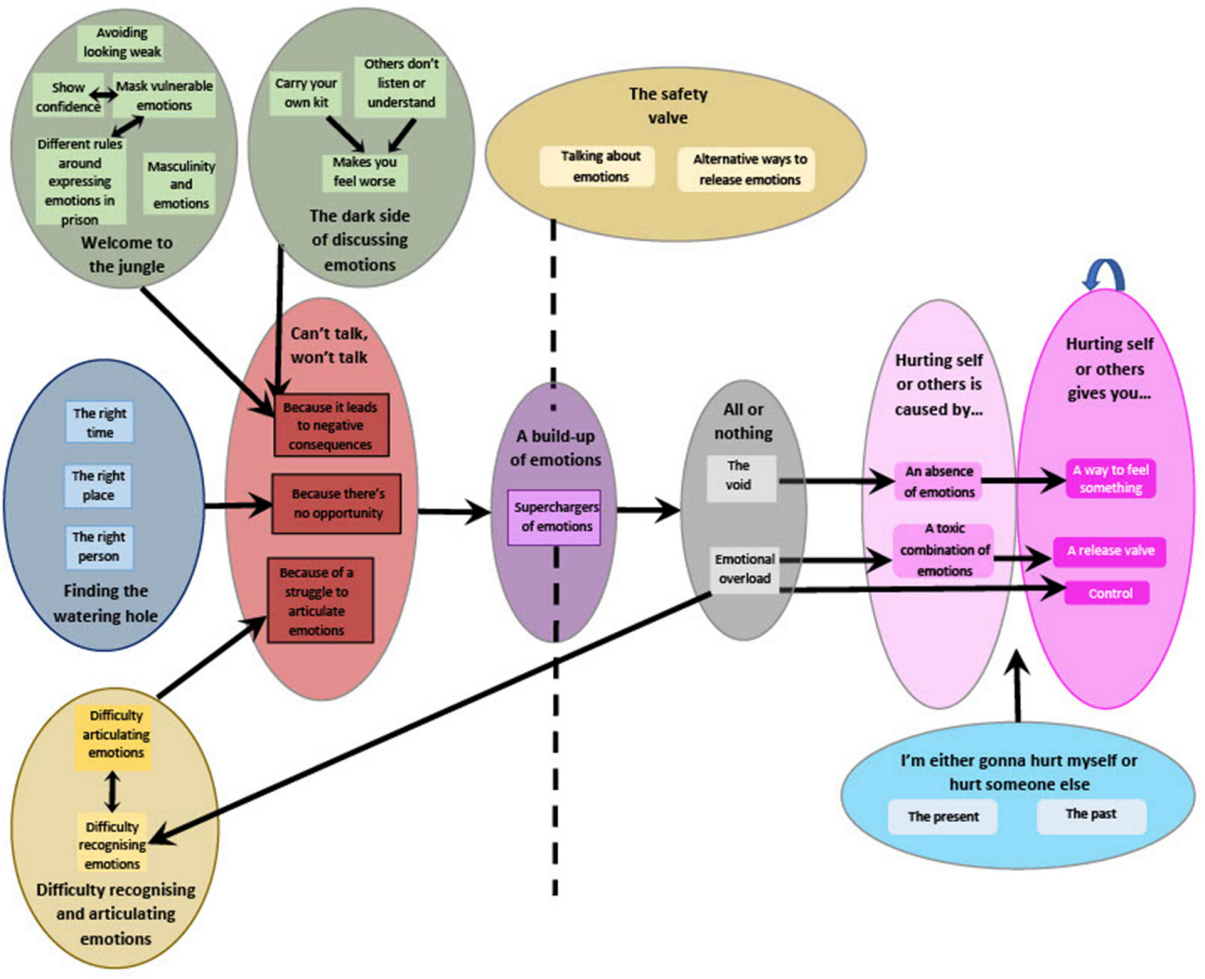
Thematic map.

In addition to this, an individual with lived experience of residing in prison (PB) was also involved in the analysis of these qualitative data. PB and LH each coded one transcript (WQ15) separately. They then met and discussed each of the codes they had assigned to the transcript. Each of PB's and LH's codes were placed onto separate colors of paper. LH and PB then considered and reflected upon each of PB's codes and decided whether any of LH's codes mapped onto these. Any revisions to existing codes were then made before the creation of an agreed coding framework. LH and PB then applied this framework independently to a different interview (WQ06), chosen due to its perceived differences from the interview with WQ15. LH and PB met to discuss areas of the transcript that did not appear to fit under the current coding framework and then revised the coding framework accordingly. LH applied the adapted coding framework to three more transcripts and then met with PB to discuss the areas that did not fit into this framework and agree upon any final revisions to the framework. The final framework was then applied to the remainder of transcripts. In the final stages of analysis, LH and PB met virtually on several occasions to organize codes into themes and to create a thematic map (Figure 1).

## Results

### Sample Characteristics

The sample were, on average, aged 34.4 years old (range: 21 to 55 years), with the majority self-identifying as male (*n* = 13) and White British (*n* = 13). The mean score on the Toronto Alexithymia Scale was 74.1 (range: 61 to 90), the mean number of self-report custodial lifetime suicide attempts was 3.5 (range: 0 to 10) and the mean number of self-report custodial violent incidents was 18.3 (range: 0 to 150). The largest number of participants did not self-report any formal psychiatric diagnoses (*n* = 7). The remaining participants self-reported the following diagnoses; personality disorder (*n* = 3,), neurodevelopmental disorder (*n* = 3), post-traumatic stress disorder (*n* = 2), schizophrenia spectrum disorder (*n* = 2), anxiety (*n* = 2), and depression (*n* = 2). The participants self-reported offenses were as follows; robbery, sexual offense, arson, drug offenses, grievous bodily harm, and murder.

### Overview of Findings

There were 11 themes and 28 subthemes which all related to a process outlined in Figure 1.

“Welcome to the jungle” and “the dark side of discussing emotions” prevented people from talking about their emotions due to perceived negative consequences. “Finding the watering hole” captured people's lack of opportunity to talk about their emotions. A “Difficulty recognizing and articulating emotions” also prevented people from talking about their emotions due to the cognitive difficulties associated with this. As a consequence, emotions were bottled up and this led to a build-up of emotions. This build-up of emotions was intensified by “superchargers of emotions” such as the use of substances and the fact that prison limits access to typical coping strategies for these emotions. This build-up of emotions could either result in the experience of an emotional overload or instead entering “the void.” Both an emotional overload and “the void” were separately associated with hurting self or others. First, participants stated that a toxic combination of emotions (“emotional overload”) could lead to hurting self and others, and that doing so provides a way to release emotions and also (re)gain a sense of control. Secondly, feeling no emotions (“the void”) could also lead to hurting self or others and doing so was perceived as a desperate attempt to feel something when currently feeling nothing at all. In addition to this emotional build up causing harm to self or others, other factors were felt to be involved in choosing whether to hurt self or others (“I'm either gonna hurt myself or hurt someone else”). Namely, the situation (“the present”) was thought to impact on this decision and also previously learnt behavior (“the past”) in relation to emotions, self-harm and violence.

Whilst the functions served by hurting self or others were seen to perpetuate this behavior, an alternative route was also identified—“the safety valve.” Here, participants acknowledged that there were alternative ways to release emotions that did not involve hurting self or others. Instead, talking about emotions to others, or finding alternate ways of expressing emotions for instance, through physical exertion or writing letters, was perceived to potentially be associated with positive outcomes.

Theme 1: Welcome to the Jungle

This theme focused on the environment that prison creates which can be experienced as hostile and masculine. As PB put it during one analysis session; you can either be an antelope, an elephant or a lion. In an environment where everyone is vying for lion status, it was therefore seen as integral to avoid looking weak (being an antelope) whilst in prison, but was acknowledged that there was a middle ground to be sought where you were neither predator nor prey (being an elephant). It was felt that showing particular emotions, such as sadness and fear, could contribute to you appearing weak. This fear of appearing weak led to participants choosing not to discuss their emotions with others.

“With prison life it's harder because it's all that bravado isn't it and that like don't wanna be seen as a weak person otherwise you get suckered. ‘Cause it does happen, like you show weakness or someone takes the piss out of you and you just let it lie then people are gonna take advantage of that situation, thinking ‘ah you're weak, I'll be able to do this, I'll be able to get away with all this with you and that’, you know what I mean? So, it is harder.” (WQ12)

Participants alluded to upbringings which outlined masculinity as a need to be the lion of the jungle, and it was acknowledged that this attitude prevailed in prison life too.

“Boys didn't cry, you know what I mean, you didn't cry back then. You didn't let anyone get anything over on you, if you got put down, if you were in a fight, you got up and you carried on fighting, or you went after them, that was the rules, you know what I mean.” (WQ09)

Participants spoke about employing several strategies to avoid looking weak whilst in prison. For instance, masking vulnerable emotions was seen as an effective way to avoid looking weak. This was achieved by maintaining a non-emotional exterior when experiencing “vulnerable” emotions internally and only expressing these emotions in private.

“Get yourself behind your door, *then* you let your emotions go, when you're on your own.” (WQ07)

**Figure 2:**
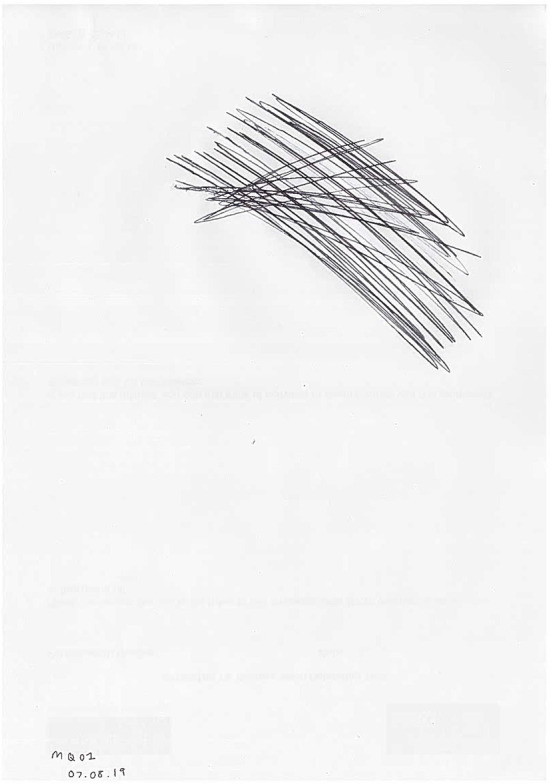
MQ01 drawing of emotions experienced before suicide attempt.

One participant attempted to convey this masking of emotions pictorially by attempting to cover the whole page black, demonstrating the “cover up” of negative emotions experienced (Figure 2). As he explained: “*It would just literally go like that until the pen's ran out or the paper's black…It's not that I don't feel emotional, it's just that I try and, like I said, I try and cover it all up. I don't want anybody else to know how I'm feeling.” (MQ01)*. Similarly, some participants created drawings which showed externally relatively “normal” faces which were at odds with the described negative feelings being experienced internally.

Indeed, masking vulnerable emotions was felt to be an inherent way of prison life, due to the different rules that existed around expressing emotions in prison. These rules, again, served the purpose of avoiding looking weak, and enforced the importance of keeping vulnerable emotions to yourself.

“Anything's seen as a weakness in prison so you can't really say to somebody ‘I'm feeling vulnerable today’… Yeah, it's more difficult in prison to talk about things than on the out, I'd have said.” (WQ06)

As well as hiding the more “vulnerable” emotions, participants simultaneously attempted to appear confident. This was achieved by demonstrating overtly the emotions and behaviors which were deemed more socially acceptable within prison. This included showing more anger, violence, and interestingly more prosocial emotions too, which were felt to make prison life easier due to attracting less conflict whilst still appearing confident.

“If you're this angry person that can have a laugh and a joke but is ready to have it whenever they need to have it, he's kind of looked on as a decent guy. Which is strange really, but I think it's just definitely to do with image.” (WQ15)

Theme 2: The Dark Side of Discussing Emotions

In this theme, participants acknowledged that discussing their emotions with others could lead to several negative consequences. These negative consequences included concerns about the recipient of the conversation who may be made to feel burdened (“carry your own kit”) or be unable to listen to or understand the conversation. Talking about emotions was also perceived to be a shameful and regretful act. Again, a concern with these negative consequences led to participants choosing not to discuss their emotions with others.

2a: Carry your own kit

There was a strong feeling amongst participants that talking about your emotions could, and would, burden others. Participants were keen to avoid this, as this often left them feeling worse than if they had not discussed their emotions with another person. Fears of burden applied to a range of contacts including friends, family and prison staff.

“I've stopped talking about my emotions because my family are ill. My brother's got cancer second stage lungs, he's terminal. My mum, she's having heart attacks and strokes all the time. So, they're my immediate family. And I wouldn't want to put my problems onto them because mine are emotional problems, they've got physical problems wrong with them and I don't want them being stressed out any more than they are. So, I'll keep my emotions in.” (MQ01)

2b: Others don't listen or understand

There was a prevalent concern amongst participants that if you chose to make yourself vulnerable and discuss your emotions with others, that others might not listen to you or understand what you were saying. This led to a sense of great frustration and, again, was perceived to make you feel worse than if you had chosen not to talk about the emotions in the first place.

“There's a big part of why should I open this can of worms up? Why should I try explain to you when you're possibly not gonna get what I mean and then I'm gonna have to tell somebody else and then explain it to somebody else and then explain it to somebody else and explain it to somebody else.” (WQ06)

“Sometimes I try and explain and explain and explain but then sometimes the people don't help. You try and explain it and they're not helping so you're just like, ‘huh this explaining's not working now’. Like, then it's violence.” (WQ11)

2c: Makes you feel worse

Participants acknowledged that discussing your emotions with others could make you feel worse. This was either due to factors associated with the other person (2a carry your own kit and 2b others don't listen or understand) or due to an internal feeling of shame or embarrassment that was associated with seeing or speaking to that person after having divulged emotions.

“Sometimes even when I do talk to staff, I just feel weird afterwards, man, I just feel awkward, man, nah it's not cool, man.” (WQ15)

Theme 3: Finding the watering hole

Participants expressed that, to talk about their emotions, the conditions had to be right. In the analogy of prison being like a jungle, participants spoke about having to find a safe environment (time, place and person) to discuss their emotions. This has been likened to finding the watering hole, where animals have to convey a certain level of trust in predators due to the necessary risk of obtaining essential water. A running thread for all three subthemes was the need to have trust in the other person. This meant waiting until this trust had been built (the right time), ensuring a confidential space was chosen to discuss emotions (the right place), and ensuring you trusted the right person with your emotions (the right person).

3a: The right time

Participants spoke about the need to find the right time to discuss emotions, both for themselves and for the recipient of the conversation. Participants spoke about the need to wait until the emotion was less intense to discuss with others to allow for a greater sense of perspective, the need to wait until a good level of trust had been instilled in the other person and also the need to consider practical implications of whether there's enough time to get into the issues in enough detail. It was also considered important to make sure that it was the right time for the other person to be hearing about your emotions, to avoid others not listening or understanding, as outlined in theme 2b “others don't listen or understand.”

“I'll sort of bite my lip until I see an officer that I *do* get on with or somebody that I *can* talk to. But by then it might be over, you know have my little crisis or whatever and then it's gone. Or they might have told me a joke and then I'm gonna tell them that ‘oh I nearly cut myself today’. You know, it's not really the time or place to do it. So, I've got to kind of wait until other people are ready to hear my problems really.” (WQ06)

3b: The right place

Participants acknowledged that there were some places in prison that were easier to discuss or express emotions than others. Whilst some felt that there was nowhere in prison you could express emotions and that they would have to wait until they were back out in the community to do so, others identified a number of places within prison where they felt more comfortable to express or discuss emotions. These included the chaplaincy, the gym and Listeners' suites (Listeners are prisoners trained by the Samaritans to give emotional and social peer support). Additionally, therapeutic community wings and wings with more elderly habitants were identified as the “right place” since these wings were seen to have eliminated the need to be a “lion.” In line with the previous theme 1 welcome to the jungle', some felt that the only place in prison to express emotions was in the privacy of their own cell.

“This wing [therapeutic community wing] is like a community. Whereas over there [main wings], it's just like a jungle over there… Well this is a community and there is good decent people who will offer you decent support and actually talk sensible to you. Whereas over there you can't get none of that.” (WQ13)

3c: The right person

Participants spoke at length about what comprised the “right person” to discuss emotions with. According to reports, the right person was not related to a particular role, for instance, friends, family or staff, but instead related to a set of essential qualities. These qualities included being compassionate, understanding, having shared experiences and knowing the other person would not share your conversation with others. Participants acknowledged that they would be unwilling to discuss emotions with anybody who did not possess these qualities. They were, therefore, unlikely to trust individuals who did not possess these qualities. This was again related to the “jungle” environment and the need to avoid looking weak.

“Just somebody being understanding really. Somebody that's going to listen and not, ‘oh right, you're unhappy’, or ‘speak to the listeners', or ‘do you want the Samaritans phone?’. Ideally don't jump with an answer that you think in your head is gonna be okay ‘cause I've had twenty odd years of self-harming and feeling like this. Sit me down if you've got time, listen to me.” (WQ06)

Participants also identified people that they would *not* talk to about their emotions. Again, this was primarily down to not trusting the other person, but also related to people being “old-school” or not receptive to discussing emotions.

“I find it quite hard to speak to my dad about emotions. ‘Cause my dad's never been like that, he's old school my dad. Even when my mum passed away, my dad didn't even show emotions, he just kept it all to himself.” (WQ12)

Theme 4: Difficulty recognizing and articulating emotions

As well as the social pressures to hide emotions, participants spoke about cognitive difficulties with both recognizing and articulating emotions. The two subthemes are intrinsically related to one another, as participants stated that struggling to recognize your emotions often led to a difficulty articulating them, and if you didn't articulate your emotions then this led to greater difficulties recognizing emotions. Both difficulties led to participants avoiding discussing their emotions with others.

4a: Difficulty recognizing emotions

Participants spoke about experiencing a struggle when trying to identify emotions in themselves, which resulted in a difficulty with labeling emotions and also a difficulty distinguishing between specific emotions. This difficulty was often attributed to the fact that people experienced lots of emotions at once, or a rapid change in emotions which made it difficult to pinpoint exactly what the person was feeling.

“It feels like I've got a jigsaw puzzle in my head but then I put the last piece in but then someone's pulled loads of pieces out all of a sudden… And then all my emotions and everything are mixed up again. Once I can just get myself straight again and get myself back up feeling good and put the last piece in and then my emotions are like ‘oh my God, what's happening?’ It's weird.” (WQ07)

“It's annoying. It's like a lot of people they understand what emotion they're feeling whereas with me I don't a lot of the time. Because obviously my depression and anxiety and all that lot when they're on their own they're alright, I can understand them. When they're all together it's a nightmare because it's like going into a boxing ring. It's a case of you never know who's going to come out on top.” (WQ05)

Participants also made a distinction between their ability to recognize an emotion in the moment, vs. at a later time upon reflection. The former of these was thought to present a greater challenge. This was related to participants being able to discuss their emotions once the emotion had passed when it wasn't so intense (3a the right time).

“Now I think about it, I don't think I was aware of it then, ‘cause when I get like that I don't think, it just happens. And I don't even understand it, I still don't understand it even though I've tried. It's hard work. But yeah, at that time I don't think I, feel like, I might *feel* like that, but I don't understand in like recognizing that it's going on in my head.” (WQ12)

4b: Difficulty articulating emotions

Very much related to a difficulty with recognizing emotions, participants also spoke about a difficulty with articulating emotions. This was felt to be both due to a lack of understanding of their own emotions, which made them difficult to explain, and also due to participants not possessing the language to discuss their emotions with others.

“It's difficult trying to find the words. It's almost like they've got a script and they want you to pick up these key words. Well tell me what the key words are and I'll read them in a sentence or something.” (WQ06)

Theme 5: Can't talk, won't talk

Due to a combination of four themes (1 welcome to the jungle, 2 the dark side of discussing emotions, 3 finding the watering hole and 4 difficulty articulating and recognizing emotions), participants spoke about how this led to them actively avoiding communication of their emotions to others. This was often framed as “bottling emotions up” or “locking” them away.

“I just don't express my feelings. Like I say, I let things bottle up… I just let things bottle up and bottle up inside and so a little thing becomes a massive thing.” (WQ09)

5a: Because it leads to negative consequences

Participants chose not to speak about emotions because it could lead to negative consequences, as outlined in the themes “welcome to the jungle” and “the dark side of discussing emotions.” Discussing emotions in prison was perceived to relate to a number of adverse outcomes including; looking weak, burdening others, others not listening or understanding, and, ultimately, it could make you feel worse. As a consequence, participants often chose not to discuss their emotions.

“I think since I was raped in here on [X] wing, I've got angry. And I can't release them emotions, speak about it. I can't open up to someone about it. ‘Cause if I open up to someone about it, I've got to relive that night. And I don't want to.” (WQ07)

5b: Because there's no opportunity

Participants reported that another reason for not discussing their emotions was due to having no opportunity to do so. This was related to the subthemes of theme 3 “finding the watering hole”—some participants felt that there was no right time, place or person to discuss emotions with in prison, and so therefore had no choice but to keep these emotions to themselves.

“There's not many people in prison that you could sit down and have a proper conversation with, a trustworthy conversation with. It's finding those very rare few who you can speak to, which takes time. But yeah, in a place like this you don't really get that much time to talk to the decent people.” (WQ06)

5c: Because of a struggle to articulate emotions

Participants spoke about avoiding discussing their emotions due to the cognitive difficulties they experienced with this, as outlined in “difficulty articulating and recognizing emotions.”

“Interviewer: Do you find it easy to put your feelings into words?” “Participant: No. Never have done… If possible, I'll avoid it and will, I don't know just try not to do it I suppose [laughs].” (WQ06)

Theme 6: A build-up of emotions

As a consequence of “bottling” emotions up, participants reported experiencing a “build-up” of emotions where gradually the “bottle” became full of emotions.

“It builds a lot of what you would call emotion up inside. But instead of taking it out on that person, you just sit there and it builds up and builds up.” (WQ02)

6a: Superchargers of emotions

In addition to this build-up of emotions, there were two factors that were identified as amplifying this build-up of emotions even more—the use of substances and a lack of access to other coping strategies. Participants spoke about both legal and illegal drugs as having an impact on mood, where emotions were either heightened or subdued. Participants also spoke about how emotions could impact on their use of substances too.

“Well, all my life I used to drink alcohol, sixteen liters of cider a day. That used to take all my emotions and my feelings away from me. But it used to make me sad and depressed as well. So that's when I tried to end my life and stuff like that, because you didn't know how to bring your emotions or feelings out properly.” (WQ02)

“I think ‘cause I've been abusing the drugs whilst I've been in prison that it's really made my emotions unstable… The drugs have basically ruined me. So, as well as suicidal thoughts, it's sometimes homicidal thoughts as well.” (WQ03)

A small number of participants also spoke about other coping strategies that they would use when living in the community to deal with the build-up of emotions, but that were not available to them in prison. This again, led to emotions either becoming out of control or numbed.

“You're just kind of stuck. You're not really entitled to anything that little bit extra in here that you can get on the out. Simply standing in the garden having a cigarette, you just can't get a change of scenery. Yeah, I'm much better on the out than on the in, I think.” (WQ06)

Theme 7: All or nothing

Participants described how this “build-up” of emotions, along with the “superchargers” of emotions could lead to one of two outcomes; you could either experience a toxic emotional overload, or you could experience “the void.”

7a: Emotional overload

In this subtheme, participants spoke about losing control of their emotions. This involved experiencing an emotional overload or feeling lots of emotions at the same time and an unpredictable change in mood. Participants also spoke about emotions “taking over” which resulted in a lack of rational thinking and often pre-empted impulsive behaviors.

“Once I feel one emotion, I feel every emotion you can possibly think of. And that's why I can't control them. Because I feel *everything* at once.” (WQ01)

**Figure 3:**
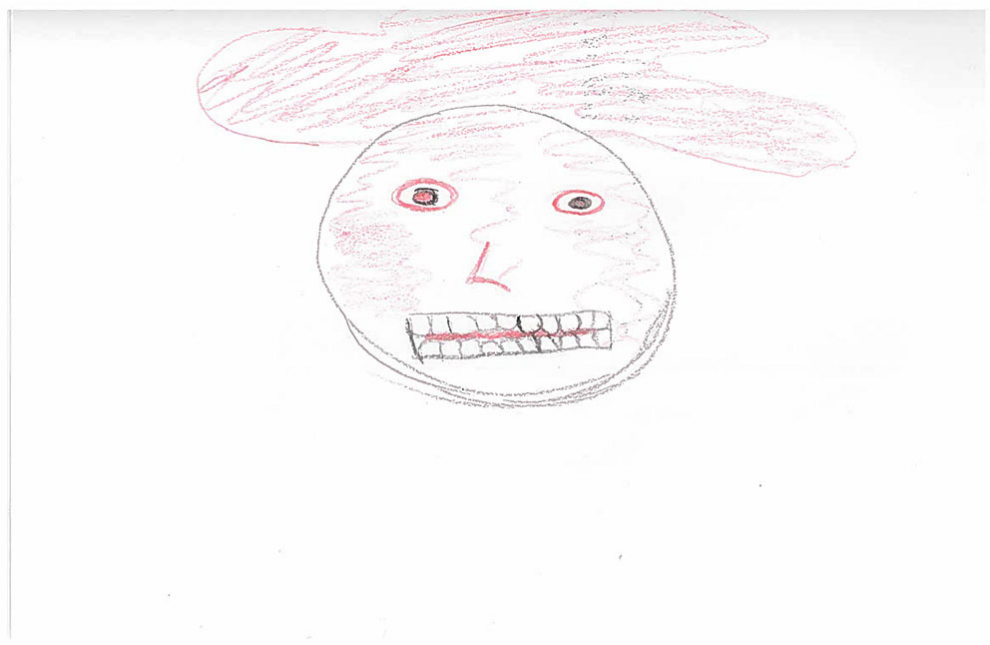
WQ10 drawing of hatred and anger.

This “emotional overload” was also present in the drawings that participants created. Notably, eleven of the drawings included more than one emotion in the same picture, suggesting it was common for participants to experience an overwhelming number of emotions at any one time. Interestingly, anger was present in all of these pictures. One participant attempted to convey the way that emotions “take over” and you lose a sense of rationality (Figure 3). As he described it, when he was angry a “red mist” would appear above his head which would cover his face and make it difficult to see what was coming next. This red mist could be popped either by lashing out (at yourself or others) or sometimes it may dissipate itself, for instance, if you had gotten angry over something inconsequential, though this was rare.

**Figure 4:**
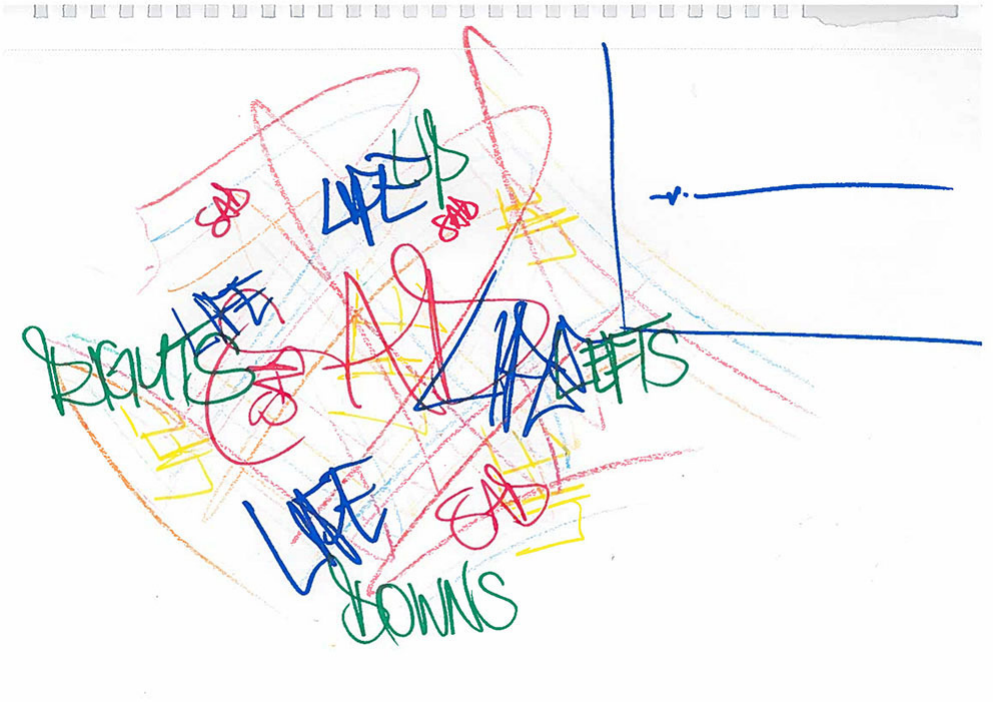
WQ11 drawing of mixed emotions and emotions experienced before suicide/violence.

Another participant attempted to illustrate the inner turmoil he experienced due to feeling an emotional overload and experiencing lots of emotions at once (Figure 4). Here, the participant conveys all the different emotions he might experience at once, as well as tying these in with life events which might be closely related to these emotions (“stressors”). The participant also acknowledged that this “mess” of emotions would usually be expressed as anger in the moment.

7b: The void

In contrast to experiencing an emotional overload, participants also spoke about experiencing an absence of emotions, or feeling “nothing at all.” Many acknowledged that this was a desirable state of emotion, due to the associated stress with highly tense emotions being eradicated. This state of emotion was therefore considered preferable to experiencing an emotional overload, and so to this end, participants often attempted to “block their emotions” out. This was sometimes achieved through use of substances. An absence of emotions was often reported as being experienced immediately prior to acts of violence or self-harm.

“I look around and I seen everyone crying and it was like, I don't understand why you's are all crying. I couldn't understand. I couldn't live what they was feeling sort of thing. It didn't feel the same for me I don't think. I just shut off my emotions up.” (MQ01)

Theme 8: Hurting self or others is caused by…

As already alluded to, the subthemes in “all or nothing” were thought to directly precede hurting self or others. Namely, hurting self or others could be caused by a combination of toxic emotions (theme 7a emotional overload) or an absence of emotions (theme 7b the void).

8a: A toxic combination of emotions

Participants acknowledged that their emotional experience that often preceded them attempting to hurt themselves, damage property or hurt others, was characterized by a toxic combination of emotions. These included mainly fear, anger and sadness.

“Interviewer: You were saying that the last time you felt this strong emotion was the last time that you self-harmed, so what exact emotion would you say you were feeling at that point?” “Participant: pretty much all of them. Stress, anger, frustration, let-down.” (WQ05)

**Figure 5:**
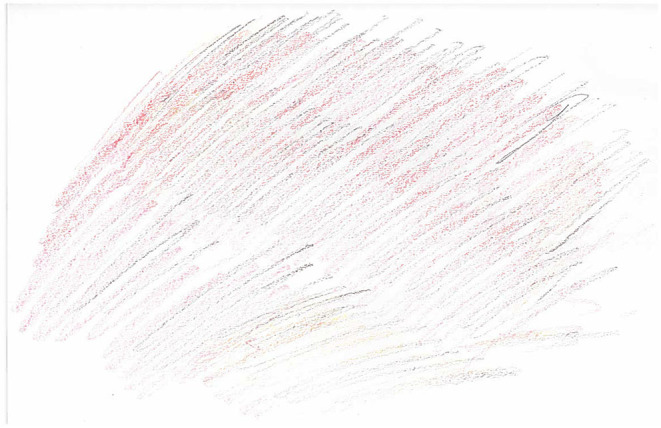
WQ10 drawing of emotions experienced before suicide attempt.

**Figure 6:**
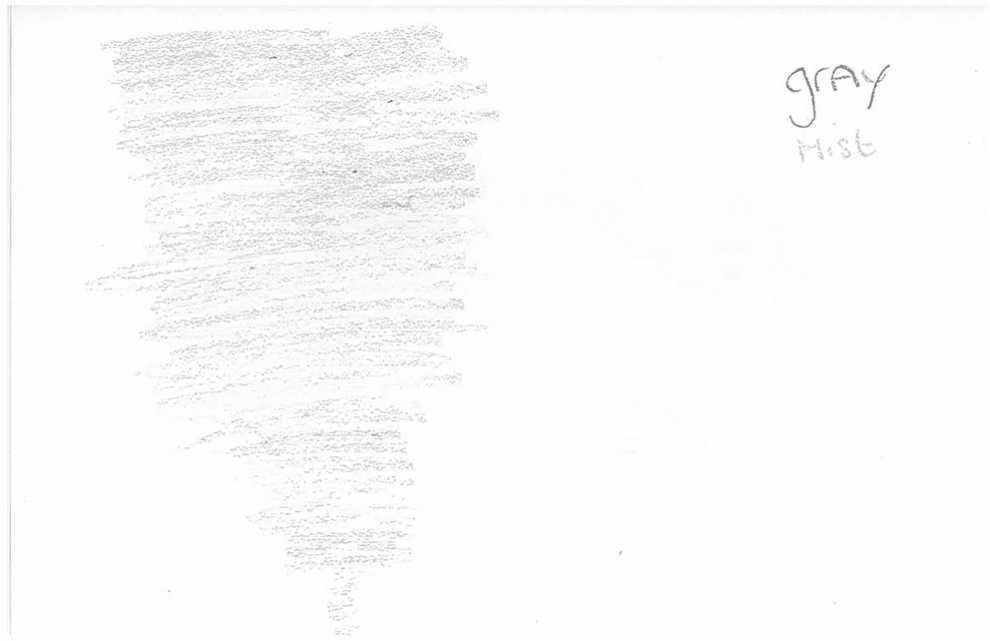
WQ10 drawing of emotions experienced before violence.

Participants also attempted to convey this in their drawings of the emotions experienced during suicide attempts or violence. For instance, one participant used color imagery to convey the different feelings he experienced when feeling suicidal which included feeling “evil” (black), “angry” (red), and “sick” (yellow; mentally and physically). These emotions were felt to be all-consuming, depicted by the fact that they cover almost the entire page (Figure 5). Furthermore, this same participant, when drawing the emotions experienced prior to violence, explained how it felt like you were in a gray whirlwind mist which made it difficult to predict what was going to happen next (Figure 6). Other participants too, described experiencing emotions in a “whirlpool”: “*I suppose in a way, it's more like a whirlpool. Just everything mixed in then it's just all going round and round.” (WQ09)*. This is again reminiscent of the “emotional overload” subtheme where participants stated that their emotions “took over,” resulting in a lack of rational thinking.

8b: An absence of emotions

As already alluded to in “the void,” participants often described experiencing an absence of emotions in the moments immediately before hurting themselves or others.

“It's like I'm empty. The only thing I can think of is like an empty bottle or something. ‘Cause at the time I don't recognize my feelings, ‘cause I start smashing things and I don't realize what I'm doing until someone tells me. So, it's like there's nothing there, there's nothing at the bottom of the bottle. The bottle's empty, there's no dregs in there.” (WQ12)

Participants again attempted to draw this absence of emotions pictorially. In WQ11's drawing (Figure 4) which shows the overload of emotions experienced, in contrast to this, the participant drew a “flatline” in a box in the top right hand corner. This “flatline” was how the participant described his emotions in the moments before he hurt himself or someone else. He also spoke about how desirable this flatline was, due to it signifying an end to internal chaotic thoughts and distressing emotions, and that in speaking to professionals he tried to explain that “flatline” is what he wanted to achieve, but that so far the only way he had found of achieving this emotional state was to hurt himself or others.

Theme 9: Hurting self or others gives you…

Hurting oneself, damaging property or hurting others was seen to provide a number of solutions to the difficulties outlined in the lead up to these behaviors. Specifically, hurting self or others provided functions to alleviate the experiences of emotional overload and not feeling any emotions at all. Because of the “rewards” that these behaviors gave people, participants explained that this perpetuated behaviors aimed at hurting self or others.

9a: A release valve

Participants frequently spoke about how hurting yourself, damaging property or hurting others served as a release from the emotional overload (theme 7a) which had built up (theme 6) as a result of not discussing emotions with others (theme 5).

“I know it's not normal for people to hit a wall until your hands are hurting or your hands are bleeding, but to me, it's my release.” (MQ01)

“When I cut myself, I'm not cutting myself to *kill* myself, I'm cutting it because I just need to release stress, man. I get *so* angry man, that I just wanna, I don't know, if I can't hurt somebody else, Ill hurt myself. And once I've cut and seen the blood coming out, you kind of breathe a bit more and it kind of just relieves that stress, man, do you know what I mean?” (WQ15)

9b: Control

Participants also spoke about how hurting yourself, damaging property or hurting others gave you a sense of control, which participants felt they lacked when experiencing an emotional overload. Participants described how their emotions “took over” in this emotional overload, and that hurting yourself or others could be a way to regain some “normality” and control over this.

“I can't control my emotions. So that's why I start doing things like cutting. Because I can't control them… Because the physical pain makes me feel in control.” (WQ01)

9c: A way to feel something

Lastly, participants spoke about how hurting yourself, damaging property or hurting others could redress an absence of emotions, as outlined in theme 7b “the void.” Such behaviors provided a way to feel something, when you don't feel anything at all.

“Just hitting something, making pain travel up my arms, just to make me feel alive. Just so I can feel something like what I imagine a normal person might be feeling if they're sad. It might hurt them inside physically or emotionally.” (MQ01)

Theme 10: I'm either gonna hurt myself or hurt someone else

Participants felt that there was a common pathway between the build-up of emotions and hurting either themselves or others—different emotions were not necessarily related to different actions. However, participants spoke about factors *outside* of emotions as being important in deciding whether to hurt themselves or hurt somebody else. Specifically, participants spoke about the current situation as having an impact on this (the present), and also about their learnt behavior (the past) in relation to suicide and violence.

10a: The present

Participants noted that the current situation often determined whether a person would engage in harming themselves or others. For instance, often self-harm was necessitated due to the person being alone in their cell and not having somebody else to hurt, or in arguments if the other person were to walk away.

“I'd take it out on myself or if somebody was there, then they'd end up getting it… If someone was there they'd get it, if they wasn't there then it'd be me that'd stand there and smash the fucking hell out at the wall.” (MQ01)

10b: The past

Participants also spoke about their upbringing and how this made them more likely to engage either in hurting themselves or in hurting others. Participants particularly highlighted how growing up around violence had taught them that this was the way to deal with their emotions.

“I think you learnt to deal [with your emotions] violently. You learn that's the way you dealt with problems, you know, you've got a problem, you strike out.” (WQ09)

Theme 11: The safety valve

Despite participants perceiving a process which led to hurting yourself or others, which included seemingly unmovable obstacles such as the prison environment and cognitive difficulties with identifying and discussing emotions, participants also acknowledged that there *could* be an alternative route which did *not* necessarily lead to hurting yourself or others. Participants were very aware of the challenge of altering the difficulties outlined in “welcome to the jungle,” “the dark side of discussing emotions,” “finding the watering hole,” and “difficulty recognizing and articulating emotions,” which also meant it was difficult to change the culture of “can't talk, won't talk” and therefore inevitably often led to a build-up of emotions. *However*, participants acknowledged that once they had experienced a build-up of emotions, instead of experiencing an emotional overload or absence of emotions which led to hurting themself or others, there was, instead, an identified “safety valve.” Here, participants outlined positive coping strategies that they had attempted to use to regain control over their emotions. Notably, these strategies included attempting to talk about their emotions to trusted others, and acknowledging that such conversations could theoretically be helpful, as well as seeking alternate ways to release emotions that did not involve harming self or others.

11a: Talking about emotions

Participants spoke about the negative consequences that had arisen as a result of *not* talking about their emotions alongside identifying perceived positive consequences that talking about emotions could bring. These perceived consequences therefore made participants *want* to be able to identify and discuss their emotions with others. Indeed, people spoke of being encouraged to speak about their emotions in the right environment, for example with mental health professionals, and the positive consequences that resulted from this.

“It's made me into a better person, letting emotions out properly, discussing things properly… I'm getting a better life out of it.” (WQ02)

11b: Alternative ways of releasing emotions

As well as attempting to talk about emotions, participants identified alternative ways that they could release the build-up of emotions that did not involve hurting themselves or others. For instance, some participants spoke about expressing their emotions through writing letters (not always shared) to loved ones, or sometimes to themselves—as a way to release emotions—but still in a private manner that avoided the fears associated with looking weak, or the negative consequences associated with the recipient of the conversation. Others used safer ways to experience a physical sensation, which did not harm themselves or others, but still served as a release for emotions such as pulling an elastic band away ‘from their wrist’ and then letting go. Others, still, used distraction techniques such as listening to music, reading a book, completing distraction “puzzles” provided by safer custody, or physical exercise to release the physical tension and stress that accompanied the “bottling up” of emotions.

“I've just got to try and do everything I can to be in a positive mood, be it watch something stupid on telly, watch a boring film, pick up a pen and do a puzzle. Just something to try and knock your mind, to try and occupy your mind, to try to think of something different. ‘Today's just a bad day’, things like that. If I've got an elastic band, I'll twang an elastic band.” (WQ06)

## Discussion

### Summary of Findings

This study aimed to explore male prisoners' subjective experiences of alexithymia and how these experiences may relate to suicide and violence. This study found that male prisoners tend to avoid discussing their emotions with others, due to a variety of individual and contextual factors. and that this could lead to a build-up of emotions. As a result, participants reported either experiencing an emotional overload or becoming void of emotions. Each of these emotional states were separately related to hurting self or others; participants did not differentiate separate pathways which led to hurting self *or* others, but identified a similar or shared pathway which led to both/either behaviors. The decision to hurt self or others was perceived to be associated with both the present situation and historical upbringing. Lastly, participants acknowledged that there was a “safety valve” which provided an alternative way of dealing with the build-up of emotions.

### Comparisons With Wider Literature

#### Negative Consequences of Discussing Emotions

This study found that the prison environment may somewhat stifle the discussion of emotions (themes 1, 2 and 5). Several previous studies have found that prisoners tend to “mask” emotions in order to deal with the physical and psychological challenges posed by the “jungle” environment of being in prison (23–27).

Previous literature has drawn a nuanced, but important distinction between “fronting” and “masking” emotions; “fronting” reflects a focus on a desired feeling which is absent whilst “masking” reflects a focus on an *un*desired feeling which is present (28). In the context of male prisoners, this has been outlined as the difference between presenting an inauthentic version of the self, based on perceived desirable emotions as opposed to concealing an authentic version of the self, which is based on undesirable emotions (29). This important distinction is reflected in the present findings through the importance to prisoners of masking vulnerable emotions whilst also simultaneously promoting emotions which suggest confidence.

Participants in the present study also reported not wanting to discuss emotions with others due to a fear of other negative consequences (theme 2). Previous research echoes the finding that prisoners were reluctant to discuss their emotions due to fears this would burden other prisoners (29) or that prison staff don't care or want to listen to prisoners problems and emotions (16).

#### Finding the Right Time, Place and Person to Discuss Emotions With

This study found it was important for prisoners to seek the right conditions to allow them to discuss emotions with others; this involved finding the right time, place and person for this conversation (themes 3 and 5). Previous research has found that prison staff perceived that prisoners were more able to communicate their emotions after the feelings had passed, as opposed to in the moment, and also that prisoners had limited time to discuss emotions with prison staff (17). Other studies have reported that prisoners struggled to discuss their emotions with loved ones over the telephone due to small time windows to make their phone calls (16).

Whilst some prisoners in the present study felt that the only place in prison to show emotions was in private in your cell, others acknowledged that there were particular places within the prison in which sharing emotions was deemed easier and more acceptable (theme 3b). Previous research has echoed this, finding that there are geographical zones in prison in which the normal prevailing rules around emotions are temporarily suspended, such as the visits room, chaplaincy, and educational classrooms and art rooms (29, 30).

Finally, it was important for participants to find the “right person” with whom to discuss their emotions (theme 3c). This finding has been echoed by others who found that although prisoners are generally unlikely to share emotions with others, there tends to exist a small network of people that they are willing to discuss their emotions with (16, 25, 29, 31). Although not evidenced in the current study, previous research has reported staff to hold a perception that prisoners would be more likely to discuss their emotions with particular staff members such as female staff and non-uniformed staff (17).

#### Difficulty Recognizing and Articulating Emotions

This study found that male prisoners reported difficulties both with recognizing and articulating emotions (themes 4 and 5). This finding is perhaps to be expected given that the eligibility criteria stated that participants must have scored highly on a scale of alexithymia. This supports previous research which suggests that the rate of alexithymia is more prevalent amongst offenders than the general population (32–35). Furthermore, previous qualitative research has found that prison staff perceive prisoners to experience difficulties with identifying, understanding and communicating feelings (17) and that female prisoners have reported difficulties with communicating their feelings to others (31).

#### A Build-Up of Emotions

As a consequence of not discussing emotions, this study found that participants experienced a “build-up” of emotions (theme 6). Previous research has also found that male prisoners experienced intense, sudden emotions as a result of bottling emotions up (17). In line with the present findings, previous research has suggested that prisoners may use drugs and alcohol as a way to subdue the intense emotions often experienced in the prison environment (31, 36, 37).

#### Hurting Self or Others Is Caused by an Absence of Emotions

Following the build-up of emotions previously outlined (theme 6), this study found that participants could then experience one of two things; an emotional numbness or an emotional overload (theme 7). Although emotional numbness was described by some as desirable, it was also acknowledged that hurting self or others was a way to “remedy” this emotional numbness (themes 8 and 9).

The anti-dissociation model of self-harm posits that individuals may experience episodes of dissociation as a result of intense emotions, and this may lead them to engage in self-harm (38). Self-harm may lead to feeling generation, whereby the individual may generate physical or emotional sensations through harming themselves (38). Support for this model in a prison environment comes from a previous study in which 60% of a sample of incarcerated adolescents reported harming themselves “to feel something even if it is pain” (39).

Emotional numbness or dissociation has also been reported to relate to suicide behavior. For instance, Shneidman (40) describes the importance of “constriction” in the suicidal process. It is purported that individuals may experience “tunnel vision” in which ordinary thoughts, emotions and responsibilities are unavailable to the conscious mind. Further, Baumeister (41) purports that prior to suicide behavior, an individual refuses meaningful thought through cognitive dissociation that eliminates all emotion, in an attempt to cease feeling negative emotions and escape pain.

Finally, emotional avoidance has been postulated to relate to violence and aggression. For instance it is suggested that an individual may experience emotional dissociation as a response to experiencing trauma, and that this state of dissociation may make them more prone to committing violent and impulsive acts (42–44). Further, it has been suggested that incarceration itself may exacerbate or even induce dissociative symptoms in individuals (45).

#### Hurting Self or Others Is Caused by an Overload of Emotions

Alternatively, instead of experiencing an emotional numbness, participants described experiencing an emotional overload (theme 7). This combination of toxic emotions was also perceived to relate to hurting self or others (themes 8 and 9).

Previous research has found that self-harm amongst prisoners may suggest an emotion regulation function of self-harm (39, 46–50), including qualitative studies in which both male and female prisoners report using self-harm to achieve emotional relief (16, 51, 52). The findings of this study also echo previous studies which have found that individuals report using self-harm as a way to regain control (53–55).

In addition to this, many theories of suicide propose that suicidal behaviors stem from the experience of painful, unmanageable emotions, from which suicide offers an escape (56, 57). Indeed, research has found that those who have attempted suicide report acute, intense affect states immediately prior to attempts (58) and also that such individuals may struggle to regulate their emotions (59).

Finally, it has also been suggested that individuals may engage in violence in an attempt to regulate (improve) their mood (60). More specifically, it has been proposed that for men, violence may function to allow men to terminate emotions deemed “vulnerable” whilst reasserting masculine identity (61, 62). In line with the findings presented here, it has been suggested that a lack of communication of emotions with others may lead to individuals relying on maladaptive ways of expressing their emotions, such as through verbal and physical aggression (60, 61).

#### I'm Either Gonna Hurt Myself or Hurt Someone Else

Though participants in the present study felt that the same emotions could lead them either to hurt themselves or others, participants noted that both the current situation and historical upbringing could impact the decision on whether to hurt self or others (theme 10). It is interesting that participants perceived a shared or similar pathway toward hurting themselves and/or others. This is in contradiction to current service provision which treats these as two separate, and distinct, behaviors. Furthermore, this contradicts previous research which has found different determinants of harm to self and others (63). Despite this, more recent research has found that a large proportion of people who harm themselves also harm others, also known as ‘dual harm (64). This theory comes from evidence which suggests that these behaviors share similar risk factors (65), which is echoed in the current findings.

One previous study supports the finding that the immediate environment may impact an individual's decision to either hurt themselves or hurt someone else. In a psychiatric ward, self-harm was more likely to take place in the evening (6–9 p.m.) in private in patients' rooms, whilst acts of aggression were common in day rooms, staff offices, hallways of the ward and in dining rooms. The reasons for self-harm were mostly unknown to staff, but aggression was most frequently provoked by denials of requests from patients or as a result of other patients' behaviors. Taken in combination, these findings suggest that factors in the immediate environment, for instance (lack of) privacy, may impact on an individual's decision whether to harm themselves or others (63).

There is a wealth of previous literature which suggests that exposure to suicide or self-harm during childhood makes an individual more likely to engage in acts which harm themselves later in life (66–70). Further, a large body of evidence exists to suggest that being exposed to violence during childhood predisposes an individual to engage in violent behavior later in life (71–73). Taken together, these studies support the finding in the present study that upbringing may impact an individual's decision to engage in acts either of harm to self or harm to others.

#### The Safety Valve

Participants in the present study perceived that there was an alternative route to harming self or others which could alleviate some of the emotional difficulties experienced whilst in prison (theme 11). Specifically, they acknowledged that talking to others as well as finding other more adaptive ways of releasing emotions, could be beneficial and lead to positive outcomes. Support for the benefit of talking about emotions comes from a pilot randomized control trial of cognitive behavioral therapy for suicidal behavior, which found that male prisoners who received the treatment achieved a significantly greater reduction in suicidal behaviors than those receiving treatment as usual (74). Further to this, research into the encouragement of harm minimization strategies which focus on providing a sensation proxy (e.g., snapping an elastic band) has found a significant reduction in self-harm incidents in women utilizing forensic services (75).

#### Individual and Contextual Factors

This study found that both individual and contextual factors were associated with participants propensity not to discuss emotions with others (themes 1,2,3,4 & 5). For instance, individual factors included an innate difficulty with recognizing and articulating emotions (theme 4). Contextual factors included the prison environment, both physical and social (themes 1 and 3), and the anticipated response to emotional disclosure from others (theme 2).

Previous research has also identified an interplay between individual factors such as difficulties identifying, understanding and expressing emotions with contextual factors such as prison norms, lack of opportunity and permission and toxic masculinity (17). This reflects previous literature which has found that, for instance, self-harm is related to intrapersonal difficulties such as alexithymia, depression and lower resilience as well as external factors such as bullying (76).

### Strengths and Limitations

Whilst it is difficult to provide a consistent explanation of the themes identified, this study provides the first qualitative exploration of male prisoner's experiences of alexithymia and how these experiences relate to suicidal and violent thoughts and/or behaviors. A strength of the paper is the involvement of people with lived experience of residing in prison, particularly their input to the qualitative analysis of data. A further strength of the study is the triangulation of data—in this case, the collection of pictorial data which can provide insight into experiences where participants are not able to rely on accurate verbal description of these.

Despite this, the study sample is limited due to all prisoners residing in the North West of England, and the majority of participants identifying as White British. Therefore, the experiences of these prisoners may differ from other populations. Furthermore, the study is limited in its exploration of experiences of violence due to participants exhibiting a social desirability response bias, whereby participants may have underreported experiences of violence due to the perception that these are socially disapproved (77). Indeed, it is known that such a response has been associated with self-report measures of anger (78). Finally, it is acknowledged that it is not possible to conclude from these data whether the findings are specific to this population or may extend to other populations. For instance, it is important to consider whether similar experiences are reported by those incarcerated with low alexithymia scores, or indeed those with high alexithymia scores that reside in the community.

### Clinical and Research Recommendations

This study has qualitatively explored male prisoners' experiences of alexithymia and how these experiences might relate to suicidal and violent thoughts and/or behaviors. Future research should aim to explore these issues in other samples such as female prisoners and young offenders, in order to explore further the extent to which the results presented here are related to notions of gender and masculinity. Furthermore, future research should aim to test this relationship using longitudinal quantitative methods in order to ascertain whether suicidal and violent behavior precedes or succeeds experiences of alexithymia. Finally, future research should aim to draw comparisons between prisoners with low and high alexithymia scores and between prisoner and community populations, in order to ascertain the potential specificity of the current study's results and the driving factors of these experiences.

The findings of this study suggest four main avenues for clinical practice. First, the findings suggest that participants do not communicate their emotions with others due to factors of the prison environment (theme 1). This suggests a need for a shift in the cultural and physical environment of prisons in order to reduce emotional distress and subsequent harmful behaviors. In particular, these findings highlight the importance of subverting the “jungle” culture that exists in prison, and to soften the pervasiveness of the gendered norms that appear to exist in prison around expressing emotions. As is alluded to in these findings, and supported by previous research (79), such a culture is attainable, since prisoners often recognize the culture in therapeutic communities as being markedly different, and more beneficial, than the “jungle” environment of prison main wings. Furthermore, several community campaigns center on the notion of encouraging men to discuss their feelings (e.g., Campaign Against Living Miserably, https://www.thecalmzone.net/), and the findings here suggest that a similar educational campaign may be of benefit in a male prison environment.

Second, it was found that participants did not discuss emotions due to a lack of opportunity to discuss these in the right time and place and with the right person (theme 3). This suggests that changes to the physical environment and staffing structure would also improve the likelihood of prisoners discussing their emotions with others. For instance, allowing male prisoners private, confidential spaces to discuss their emotions, and enabling these to be used at times chosen by the prisoner would likely improve the chances of prisoners discussing their emotions with others, which in turn could help to prevent suicide and violence in prison. In addition, it's important that prison staff, in all roles, are encouraged to show qualities such as compassion and understanding (theme 3c), as this, too, could encourage prisoners to confide in staff about their emotions. Such an approach could be encouraged through providing training to staff to provide the necessary understanding and skills to be able to approach emotional difficulties with compassion and understanding. An example of one such approach is the GRACE model of compassion which has been encouraged in nurses as a way to develop and practice compassion (80).

Third, the study found that in addition to external pressures, some people may not discuss their emotions due to an internal difficulty with recognizing and articulating emotions (theme 4). This suggests a need to provide tailored individual support to those prisoners who exhibit difficulties in recognizing and articulating their emotions. In addition to the environmental pressures which inhibited discussion of emotions, participants also identified innate cognitive difficulties with recognizing and articulating emotions. Thus, a two pronged approach to intervention is needed to tackle both internal and external risk factors for suicide and violence, in line with the currently enforced approach to suicide prevention in prisons which tends to focus on primary strategies that target environmental factors and secondary strategies which focus on ways to intervene with offenders considered to be at high risk of suicide (81). As such, there is a need for interventions that directly reduce the severity and impact of alexithymia. There is some evidence to suggest such an approach is both effective and acceptable to a prisoner population (82). It is therefore recommended that male prisoners be routinely screened for levels of alexithymia, and that those who score above a certain threshold are given timely access to psychological intervention aimed at reducing alexithymia. According to the findings of this study, such an effort may, in turn, reduce rates of suicide and violence amongst prisoners.

Lastly, participants in this study identified a “safety valve” which could prevent harmful behaviors (theme 11). This indicates that prison staff should place an emphasis on alternative ways that prisoners can release their emotions. Such psychosocial interventions could include encouraging harm minimization strategies such as snapping an elastic band against the wrist, encouraging use of the gym particularly at the time of intense emotions being experienced, offering distraction packs and meaningful work and education and encouraging the use of letter writing as a way to express emotions. Whilst some of these activities are already utilized in male prisons, the current study suggests that if used effectively and at the right time, these relatively brief and inexpensive interventions can help to reduce the rates of suicide and violence amongst male prisoners.

## Data Availability Statement

The datasets presented in this article are not readily available because of potentially identifiable information in the raw data. Requests to access the datasets should be directed to Laura Hemming, laura.hemming@manchester.ac.uk.

## Ethics Statement

The studies involving human participants were reviewed and approved by NHS England Research Ethics Committee. The patients/participants provided their written informed consent to participate in this study. Written informed consent was obtained from the individual(s) for the publication of any potentially identifiable images or data included in this article.

## Author Contributions

LH conducted all interviews and wrote the first draft of the manuscript. LH, PB, GH, and JS were all involved in analysis. GH, JS, DP, and PB wrote sections of the manuscript. All authors contributed to manuscript revision, read and approved the submitted version.

## Conflict of Interest

The authors declare that the research was conducted in the absence of any commercial or financial relationships that could be construed as a potential conflict of interest.
