# Supplementary material for: Words Don't Come Easy: How Male Prisoners' Difficulties Identifying and Discussing Feelings Relate to Suicide and Violence
Source: Front Psychiatry. 2020 Dec 10;11:581390. doi: 10.3389/fpsyt.2020.581390 (PMC7758448; doi:10.3389/fpsyt.2020.581390)
Supplement: Supplementary file 1 [file Data_Sheet_1.PDF]

MANCHESTER 1824
The University of Manchester

Medical Research Council MRC

How do suicidal and aggressive prisoners manage emotion?

Prisoners topic guide

1. Can you tell me what you think I mean when we talk about emotions / feelings?
    - *Probe: distinct from drives (hunger, sex)? Distinct from thoughts? Distinct from physical states?*

When I say emotions / feelings, what I'm talking about is when people feel e.g. sad, happy or angry. These are feelings that cannot be helped and are experienced by lots of people. These are different from thoughts that we might have or behaviours that we might do. An example of a feeling is sadness. When people feel sad they might become overwhelmed with things. They might cry a lot. They might become withdrawn. Does this make sense?

2. Have you experienced any strong emotions lately? Can you tell me about a recent time?
    - *Probe: upset, happy, worried, disgusted, shame, angry, love*
    - a. How did you know you were experiencing a strong emotion? What was the first thing you noticed? When did you notice this?
    - b. What is it like to feel like this?
    - *Probe: physical reactions, thoughts*
    - If you could draw these emotions, what would they look like? Would you like to have a go at drawing these for me?
3. How did you express these emotions? How did you feel about expressing these emotions?
    - *Communication – who with? How did others respond to this? What thoughts were you communicating? Did you put your feelings into words – how did you find this? When did you express them (at the time or on reflection?)*
    - *Does prison impact on how / if you talk about your emotions?*
    - *How did you respond / react to these feelings? What did you then to? Behaviour – violence, self-harm, suicide, isolation, argue. Was this something you had a choice over or did it just happen?*
    - *Did you feel comfortable? Overwhelmed?*
4. How did others in your home show their feelings? Were some emotions shown more often than others? What do you think you learned from this?
    - *Probe: positive as well as negative learning*
5. You recently filled in some questionnaires with me – do you remember? I wondered if we could talk about your responses?
    - a. Elaboration of items on TAS-20
6. Can you tell me about the most recent time that you thought about or engaged in suicidal / aggressive behaviour?
    - a. When was it? What sorts of things were happening for you at this time?
    - b. What sorts of emotions were you experiencing? Can you describe these to me?
        - *Sadness, anger, frustration, hopelessness*
    - c. Do you feel that your emotions played a role in engaging with these thoughts / behaviour? In what way?
7. What impact do you think your emotions have on your thoughts / behaviour around suicide /aggression?

Version 10.4, 19.07.19
IRAS ID: 222430

a. Do you have a theory for how they link together?
b. Which specific feelings / emotions contribute? Any emotions you *don't* feel which might contribute?

Wind-down questions (2-3 to be asked)

Thank you for answering all of these difficult questions – I know this is not that easy sometimes. Can we now move onto some more everyday questions?

8. Is there anything that helps you when you're feeling emotional?
9. Where have you found a good place to show your feelings? What do you do here?
    - *E.g. gym, chaplaincy, association*
10. How have you found it answering these questions today?
11. I know that this session might have been upsetting for you today. Is there anything that you can do for the rest of the day to make you feel a little better?
    - *E.g. read a book, gym, television programmes?*
12. Have you got any hobbies? *Interests, sports, TV programmes*
13. What have you got coming in canteen?
14. Any visits booked with family / friends?
